# Bicycle Set-Up Dimensions and Cycling Kinematics: A Consensus Statement Using Delphi Methodology

**DOI:** 10.1007/s40279-024-02100-6

**Published:** 2024-09-20

**Authors:** Jose Ignacio Priego-Quesada, Marco Arkesteijn, William Bertucci, Rodrigo R. Bini, Felipe P. Carpes, Fernando Diefenthaeler, Sylvain Dorel, Borut Fonda, Anthony A. Gatti, Wendy Holliday, Ina Janssen, Jose L. López Elvira, Geoffrey Millour, Pedro Perez-Soriano, Jeroen Swart, Paul Visentini, Songning Zhang, Alberto Encarnación-Martínez

**Affiliations:** 1 Research Group in Sports Biomechanics (GIBD), Department of Physical Education and Sports, Faculty of Physical Activity and Sport Sciences, University of Valencia, C/Gascó Oliag, 3, 46010 Valencia, Spain; 2 Red Española de Investigación del Rendimiento Deportivo en Ciclismo y Mujer (REDICYM), Ontinyent, Spain; 3 Department of Life Sciences, Aberystwyth University, Aberystwyth, UK; 4 Laboratoire Performance Métrologie Santé Société (PSMS EA 7507), Université de Reims Champagne Ardenne (URCA), Reims, France; 5 La Trobe Rural Health School, La Trobe University, Bendigo, Australia; 6 Applied Neuromechanics Group, Laboratory of Neuromechanics, Federal University of Pampa, Uruguaiana, RS Brazil; 7 Laboratório de Biomecânica, Centro de Desportos, Universidade Federal de Santa Catarina, Florianópolis, Brazil; 8 Nantes Université, Movement-Interactions-Performance, MIP, UR 4334, 44000 Nantes, France; 9 Faculty of Health Studies, University of Primorska, Izola, Slovenia; 10 Department of Radiology, Stanford University, Stanford, USA; 11 Division of Sports and Exercise Medicine, HPALS Research Center, University of Cape Town, Boundary Rd, Cape Town, South Africa; 12 Sport Science and Innovation, Sportcentrum Papendal, Arnhem, The Netherlands; 13 Department of Sport Sciences, Sports Research Centre, Miguel Hernandez University of Elche, Elche, Spain; 14 Laboratoire de technologies & d’innovation pour la performance sportive, Université du Québec à Trois-Rivières, Trois-Rivières, QC Canada; 15 La Trobe Sport and Exercise Medicine Research Centre, School of Allied Health, Human Services and Sport, La Trobe University, Melbourne, Australia; 16 Department of Kinesiology, Recreation and Sport Studies, The University of Tennessee, Knoxville, TN USA

## Abstract

Bicycle set-up dimensions and cycling kinematic data are important components of bicycle fitting and cyclist testing protocols. However, there are no guidelines on how bicycles should be measured and how kinematic data should be collected to increase the reliability of outcomes. This article proposes a consensus regarding bicycle set-up dimensions and recommendations for collecting cycling-related kinematic data. Four core members recruited panellists, prepared the document to review in each round for panellists, analysed the scores and comments of the expert panellists, reported the decisions and communicated with panellists. Fourteen experts with experience in research involving cycling kinematics and/or bicycle fitting agreed to participate as panellists. An initial list of 17 statements was proposed, rated using a five-point Likert scale and commented on by panellists in three rounds of anonymous surveys following a Delphi procedure. The consensus was agreed upon when more than 80% of the panellists scored the statement with values of 4 and 5 (moderately and strongly agree) with an interquartile range of less than or equal to 1. A consensus was achieved for eight statements addressing bicycle set-up dimensions (e.g. saddle height, saddle setback, etc.) and nine statements for cycling kinematic assessment (e.g. kinematic method, two-dimensional methodology, etc.). This consensus statement provides a list of recommendations about how bicycle set-up dimensions should be measured and the best practices for collecting cycling kinematic data. These recommendations should improve the transparency, reproducibility, standardisation and interpretation of bicycle measurements and cycling kinematic data for researchers, bicycle fitters and cycling related practitioners.

**Supplementary Information:**

The online version contains supplementary material available at 10.1007/s40279-024-02100-6.

## Key Points

**Table:** 

Consensus was achieved for eight statements addressing bicycle set-up dimensions and nine statements on cycling kinematic assessment.
The recommendations will improve transparency, reproducibility, standardisation and interpretation of bicycle measurements and cycling kinematic data.
These guidelines are dedicated to assisting researchers, bicycle fitters and cycling-related practitioners.

## Introduction

Cycling is a popular physical activity with numerous health benefits [1], but many cyclists are exposed to non-traumatic injuries (52–65%) [2, 3], with a suboptimal position during cycling suggested as one of the possible causes [4–6]. Bicycle fitting has been utilised to improve comfort, reduce pain and potentially mitigate the risk of non-traumatic injuries [6, 7]. Bicycle fitting has gained popularity in the cycling community. Still, there is a great variety of sources of information on which the practice of bicycle fitting is based (e.g. websites, books, research papers, etc.), as well as the methods and processes used [8]. There is a lack of evidence-based guidelines on how bicycle set-up dimensions should be measured, which may have resulted in inconsistent and varied bicycle fitting protocols. One example is the measurement of bicycle saddle height, which has a range of different methods reported (e.g. taken from the top edge of the saddle, the nose or from the broadest section of the saddle measured in line with the seat tube to the pedal spindle or the bottom bracket and/or adding the crank length), despite being one of the variables most studied in literature [9, 10].

Although a bicycle could be fitted based on anthropometrical measures when equipment or expertise is unavailable [11], research evidence suggests that bicycle fitting should be informed and guided by kinematic data obtained while cycling [10, 12, 13]. While standard recommendations are available for other forms of kinematic assessment [14], such as walking, running [15] and foot mechanics [16], no recommendations are available for cycling. One example of a limitation in cycling kinematic assessment is the inaccessibility of the anterior–superior iliac spine while cycling, which then affects the options for landmarks from which to model the pelvis [17]. The lack of consensus concerning the collection of cycling kinematic data and bicycle measurements limits the validity of the data and the ability to compare between studies. Guidelines for bicycle measurement and collecting kinematic data are seen to be essential.

There are an increasing number of professional bicycle fitting services based on kinematics, utilising two-dimensional (2D) or three-dimensional (3D) data [13, 18]. In addition to the technical challenges in implementing these measurements [18], exercise characteristics can influence kinematic outcomes [19–21]. Previous research has shown that exercise intensity [20], cadence [21] and fatigue [22, 23] can influence pedalling kinematics, but these often vary substantially across studies. Some of the authors in the current paper pointed out these challenges in an editorial that suggested the necessity of creating consensus regarding critical elements for assessing body position during cycling [18]. We, therefore, considered that a consensus statement using the Delphi method can assist researchers and cycling related practitioners in obtaining bicycle set-up dimensions and cycling kinematic data. The Delphi method consists of a structured group process to survey expert opinion and reach a group response [24]. This paper aims to obtain consensus about bicycle set-up dimensions and recommendations for performing cycling kinematic assessments.

## Methods

### Participants

Four core members (J.I.P.Q., R.R.B., F.P.C. and A.E.-M.) initiated the study, being responsible for recruiting the panellists, preparing the document to review in each round for panel members, analysing the scores and comments of panellists and reporting the decisions, as well as communicating with panellists. Core members did not participate as panel members, and were affiliated with institutions in Spain (J.I.P.Q. and A.E.-M.), Australia (R.R.B.) and Brazil (F.P.C.). None of the core members and panellists had conflicts of interest relevant to participation in the study.

Panellists were selected based on their expertise in cycling kinematics and/or bicycle fitting studies. The inclusion criteria were to have at least five publications about cycling biomechanics in journals indexed in the Web of Science or three publications about cycling kinematics in journals with impact factors in the first or second quartile. A total of 22 experts were invited, and 14 agreed to participate as panel members in the study. Two experts declined the invitation because they were too busy to participate, one declined due to a lack of interest in the topic and five did not respond. They received an information document outlining the study and the methodology with consent indicated by completion of the Delphi survey. Of the 14 panellists (mean ± standard deviation age of 46 ± 9 years old, minimum age 31 years old and maximum age 63 years old), 13 were currently working in academic and/or research institutions, and 6 of these experts (43%) work in a bicycle fitting service. The panellists were affiliated with institutions from the USA (*n* = 3; 21%), Spain (*n* = 2; 14%), South Africa (*n* = 1; 7%), France (*n* = 2; 14%), Canada (*n* = 1; 7%), Brazil (*n* = 1; 7%), Slovenia (*n* = 1; 7%), United Kingdom (*n* = 1; 7%), the Netherlands (*n* = 1; 7%) and Australia (*n* = 1; 7%). A search on the Scopus database was performed on 29 March 2023 to ensure that panellists had published a median of 6 (range 3–24) full peer-reviewed articles related to cycling kinematics and bicycle fitting, and they had a median H index of 11 (range 3–25).

### Procedures

A Delphi procedure was applied as previously described [24, 25]. For this procedure, panel expert evaluation, judgment, phrasing and scoring were completed independently for each round. Core members wrote an initial document (Supplementary file) with a list of statements to be scored and commented on by the panel members. Each of these statements also included research background information to understand the reason for each statement. Moreover, a five-point Likert scale (strongly agree, moderately agree, neutral, moderately disagree, strongly disagree; from 5 to 1, respectively) was used to rate each statement, and an open box included comments from the panellists. They could also propose a new statement for the next round. The panellists were encouraged to support their opinions with evidence whenever possible. The consensus was reached when more than 80% of the panel members scored the statement with values of ‘4’ (moderately agree) or ‘5’ (strongly agree), with an interquartile range less than or equal to 1 [25]. However, considerable modifications were made to improve transparency whenever the consensus was reached based on panellist comments, and a re-evaluation was performed.

Three rounds were performed. In every round, all panellists had the opportunity to comment on the statements and suggest possible rephrasing. The panellists had 15 days to respond to each round, and all communications were conducted by electronic mail. For the second and third rounds, the document included each statement: the statement proposed in the previous round, the quantitative analysis of the first round (percentage of answers for each point of the Likert scale and interquartile range), the qualitative analysis (a summary of all comments), the core members' conclusion, whether the statement was accepted or not and the new statement proposed. The decision made in each round for each statement comprised seven distinct actions [26]: (1) modify (the statement was substantially modified), (2) rephrase (to improve understanding without changing the meaning), (3) divide (one statement being split in two or more statements) (4) join (the statement resulting from merging two or more previous statements), (5) exclude (the statement was excluded from the consensus), (6) include (a new statement was included as suggested by a panel member) and (7) approval (when consensus was reached on a statement).

### Equity, Diversity and Inclusion Statement

The core members attempted to recruit panellists of both sexes and from around the world, including researchers in developing countries. However, of the 14 panellists, there were only 2 women, and 12 panellists were Caucasian. The established inclusion criteria limited the ability to select more women and people from other communities, which may have limited the generalisability of our results.

## Results

**Fig. 1:**
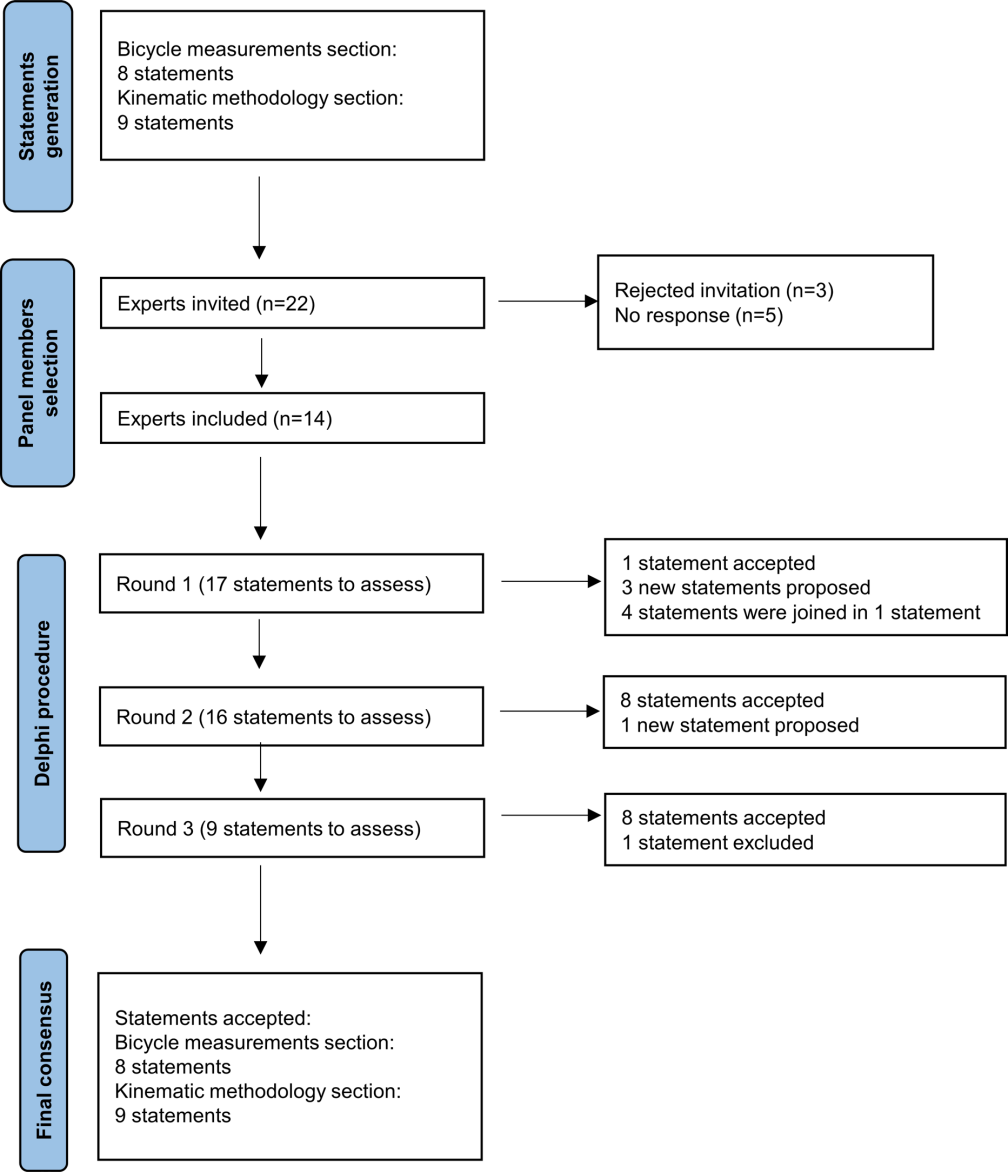
Flowchart of the process

Figure 1 shows the flowchart of the Delphi process with the results for the three rounds of evaluation.

Table 1 shows the results and decisions of each round. Decisions in the first round included modification (11 statements; 65% of the statements), joining (4 statements; 24%), rephrasing (1 statement; 6%) and approval (1 statement; 6%). Although most of the statements met the approval criteria (12 statements; 71%), only 1 was approved in the first round (item 9), and the others were revised based on panellists’ comments and were re-evaluated in the second round. Moreover, considering comments from panellists, three new statements were created for the second round: kinematic knee flexion at the bottom of the revolution (item 19), angle definitions (item 20) and foot position on the pedal (item 21). Statements showing a higher level of disagreement between panel members were those related to the saddle setback (item 4), the diagonal distance between the handlebar and saddle (item 8) and the offset by the individual’s standing posture for bicycle fitting (item 13).

**Table 1:** Results of the panellist’s evaluation ( of scores of five-point Likert scale for each item and interquartile) through the three rounds with core panel decisions for each statement

Statement	Round 1 (from 11 October 2021 to 30 October 2021)							Round 2 (from 4 March 2022 to 20 March 2022)							Round 3 (from 2 March 2023 to 24 March 2023)						
	SD	MD	N	MA	SA	IQ ≤ 1	Decision	SD	MD	N	MA	SA	IQ ≤ 1	Decision	SD	MD	N	MA	SA	IQ ≤ 1	Decision
Bicycle set-up dimensions																					
1. Bicycle	0	0	7	14	79	Yes	Modify	0	0	0	14	88	Yes	Approve	–	–	–	–	–	–	–
2. Tools	0	0	0	7	93	Yes	Modify	0	0	0	29	71	Yes	Modify	0	0	0	21	79	Yes	Approve
3. Saddle height	7	14	0	50	29	Yes	Modify	0	7	0	36	57	Yes	Approve	–	–	–	–	–	–	–
4. Saddle setback	14	21	14	36	14	No	Modify	0	7	0	43	50	Yes	Modify	7	0	0	21	71	Yes	Approve
5. Seat tube angle	7	0	14	7	71	Yes	Modify	0	7	7	50	36	Yes	Modify	0	10	0	30	60	Yes	Approve
6. Crank length	0	0	0	7	93	Yes	Modify	0	0	0	0	100	Yes	Approve	–	–	–	–	–	–	–
7. Vertical handlebar	0	0	0	43	57	Yes	Modify	0	0	0	29	71	Yes	Approve	–	–	–	–	–	–	–
8. Horizontal handlebar	29	0	7	29	36	No	Modify	14	0	7	14	64	Yes	Modify	7	0	0	7	86	Yes	Approve
Kinematic methodology																					
9. Conditions	0	0	0	21	79	Yes	Approve	–	–	–	–	–	–	–	–	–	–	–	–	–	–
10. Dynamic assessment	0	14	0	21	64	Yes	Modify	0	0	0	36	64	Yes	Approve	–	–	–	–	–	–	–
11. Kinematic method	0	0	0	14	86	Yes	Join 12	–	–	–	–	–	–	–	–	–	–	–	–	–	–
12. 2D versus 3D	0	0	0	29	71	Yes	Rephrase	0	0	0	7	93	Yes	Approve	–	–	–	–	–	–	–
13. Standing posture	8	15	31	8	39	No	Modify	0	8	15	23	54	Yes	Modify	0	0	7	36	57	Yes	Approve
14. 2D methodology	0	0	0	21	79	Yes	Join 17	–	–	–	–	–	–	–	–	–	–	–	–	–	–
15. 3D methodology	0	0	0	21	79	Yes	Join 17	–	–	–	–	–	–	–	–	–	–	–	–	–	–
16. Other methodologies	0	0	0	14	86	Yes	Join 17	–	–	–	–	–	–	–	–	–	–	–	–	–	–
17. Methodology/analysis	–	–	–	–	–	–	–	0	0	0	14	86	Yes	Approve	–	–	–	–	–	–	–
18. Full body position	0	8	8	54	31	Yes	Modify	0	0	8	14	79	Yes	Approve	–	–	–	–	–	–	–
19. Knee flexion bottom	–	–	–	–	–	–	Include	0	0	8	21	71	Yes	Modify	0	0	0	21	79	Yes	Approve
20. Angle definitions	–	–	–	–	–	–	Include	7	21	0	21	50	No	Modify	0	14	7	57	21	Yes	Exclude
21. Foot position	–	–	–	–	–	–	Include	0	7	21	21	50	No	Modify	0	0	0	15	85	Yes	Approve
22. Sample characterisation	–	–	–	–	–	–	Include	–	–	–	–	–	–	–	0	0	0	21	79	Yes	Approve

*SD* strongly disagree, *MD* moderately disagree, *N* neutral, *MA* moderately agree, *SA* strongly agree, *IQ* interquartile

In the second round, most of the statements improved their percentages of responses with ‘strongly agree’ and ‘moderately agree’ levels and met the approval criteria (13 items; 81%). However, this round did not approve five of these (31%) statements. Thus, the decisions in the second round included modification (eight statements; 50%) and approval (eight statements; 50%). They were edited considering panellists’ suggestions and re-evaluated in a third round. A new statement was created for the third round consisting of sample characterisation (item 22). Statements that had the highest level of disagreement among panel members were two of the statements with high levels of disagreement in the first round (item 8—the diagonal distance between the handlebar and saddle—and item 12—the offset by the individual’s standing posture for bicycle fitting) and two of the newly included statements related to angle definitions (item 20) and foot position on the pedal (item 21).

Decisions in the third round included approval (eight statements; 89%) and exclusion one statement; 11%). Except for the statement about the angle definitions (item 20), which was excluded due to a high level of disagreement in the second round, the other eight statements improved their scores after the third round.

The initial proposal for item 20 suggested adopting the International Society of Biomechanics (ISB) conventions for defining angles in 3D analysis. While recognising the need for further investigation into angles related to the spine and trunk due to their complex degrees of freedom, a proposal was also made for measuring angular kinematics in 2D. However, due to inconsistencies in using different joint angles (e.g. ankle, shoulder and elbow) versus supplementary angles (e.g. knee and hip), practical issues with using the posterior superior iliac spine (PSIS) and anterior superior iliac spine (ASIS) to analyse hip angles and discrepancies observed between dynamic measurements and static measurements obtained with different instruments, the item was ultimately rejected.

Therefore, the final consensus was achieved for eight statements for bicycle measurements (Table 2) and nine statements for kinematic methodology (Table 3).

**Table 2:** Statements that obtained a consensus for bicycle set-up dimensions after the Delphi procedure

**Fig. 2:**
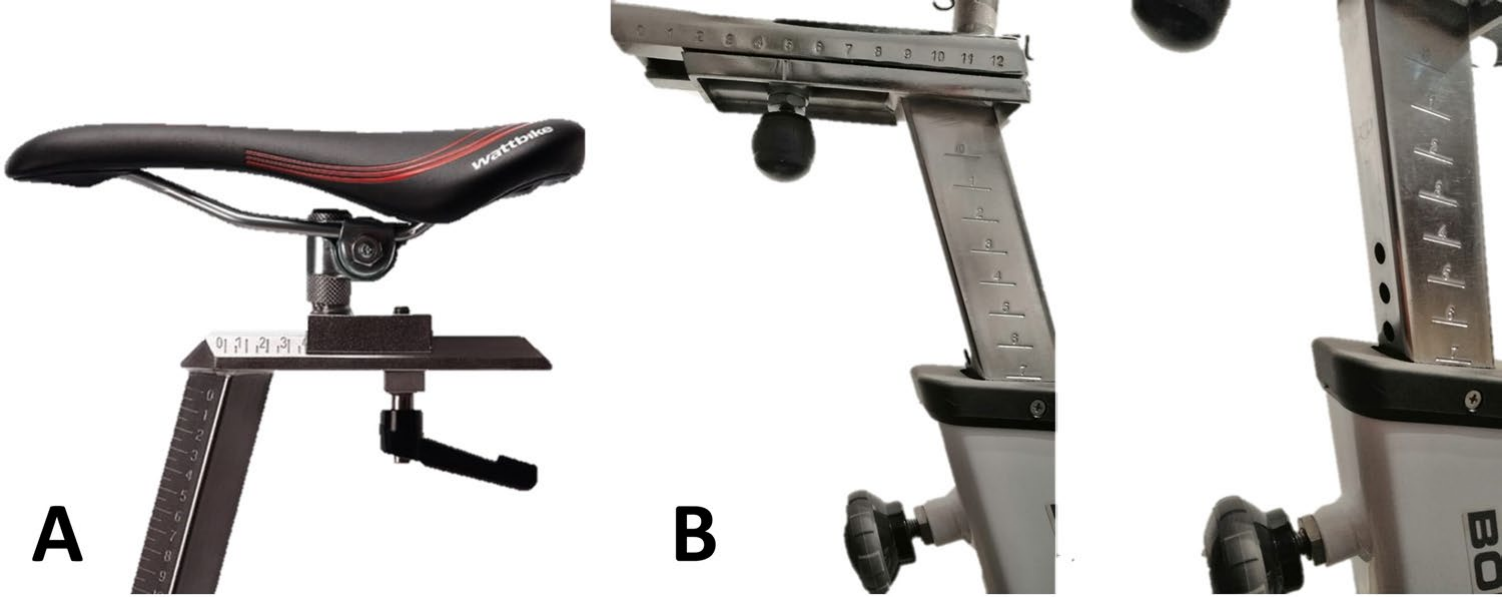
Example of saddle mechanisms adjustment. **A** Continuous adjustments system (adapted from https://wattbike.com/). **B** Discrete adjustment system (original image, Bodytone Monster model; Bodytone International Sport SL, Murcia, Spain)

**Fig. 3:**
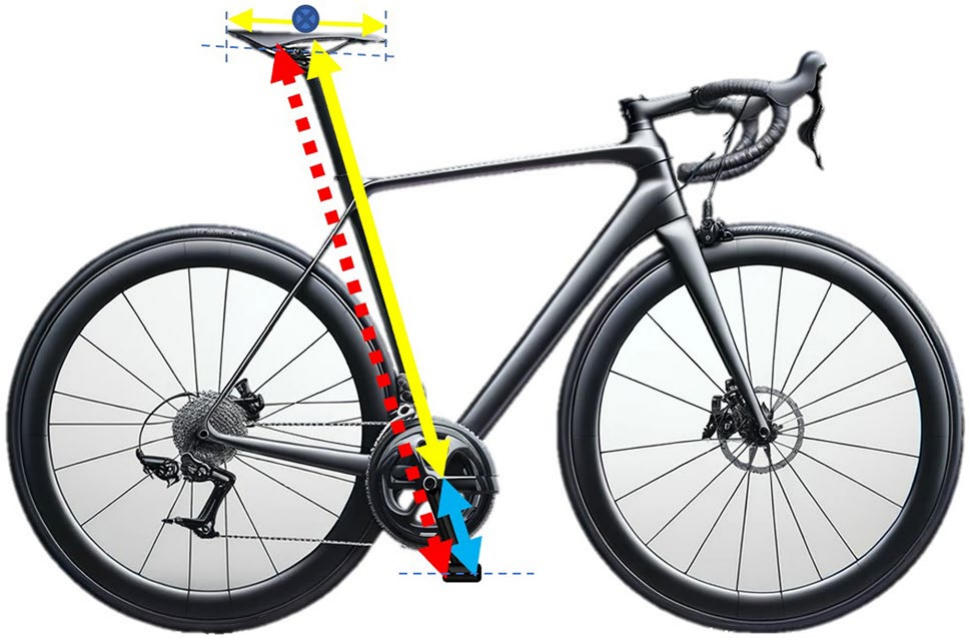
Proposed saddle height measurement. The red dashed line represents the sum of the yellow (from the bottom bracket to the centre of the saddle) and blue lines (crank length)

**Fig. 4:**
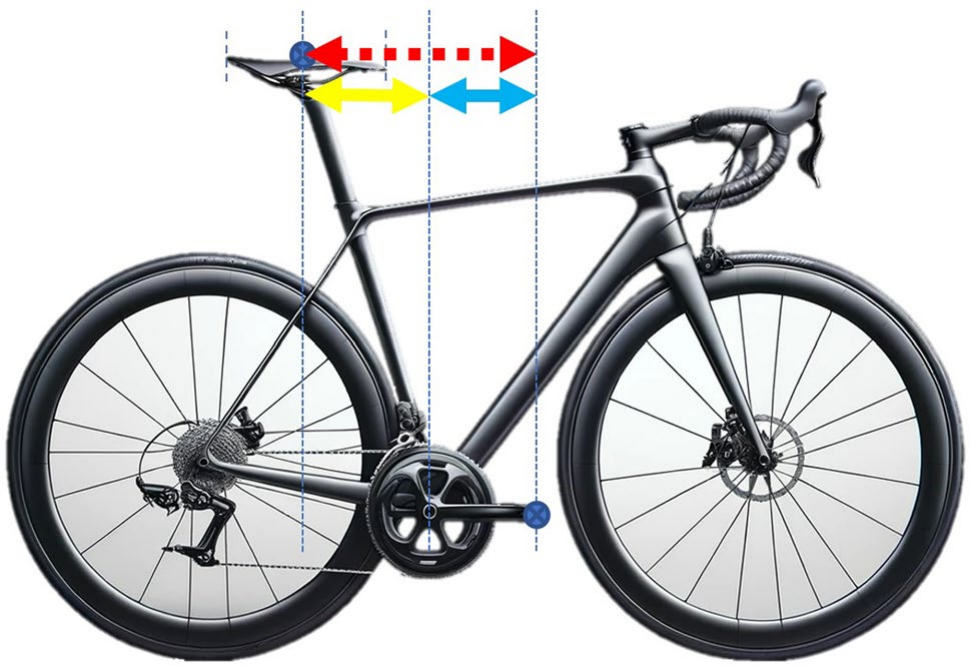
Proposed saddle setback measurement. Red dashed line represents the sum of the yellow (horizontal distance from bottom bracket to the centre of the saddle) and blue lines (crank length)

**Fig. 5:**
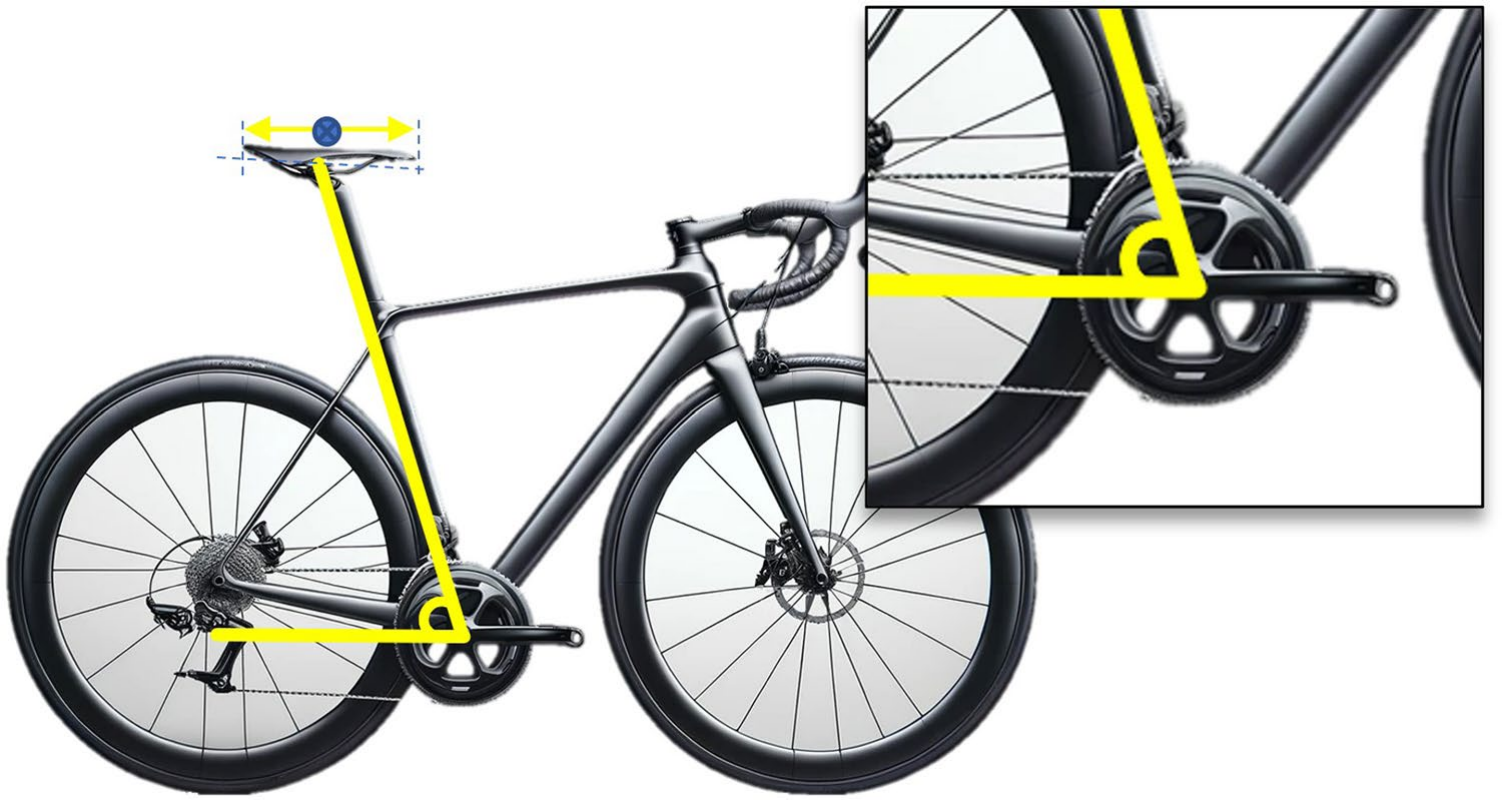
Effective seat tube angle

**Fig. 6:**
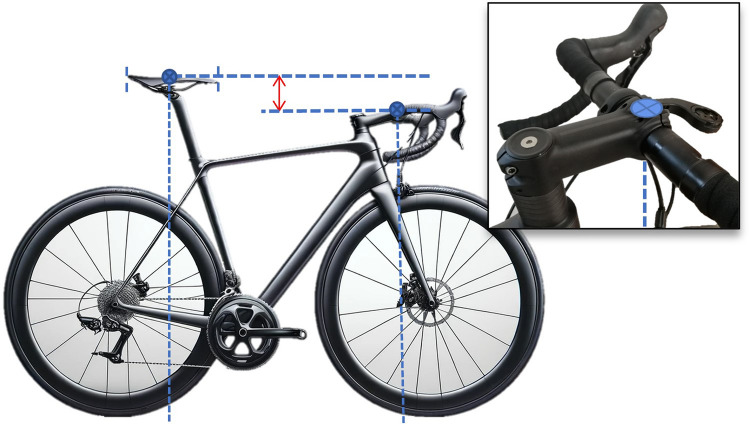
Proposed vertical difference between handlebar and saddle height measurement

**Fig. 7:**
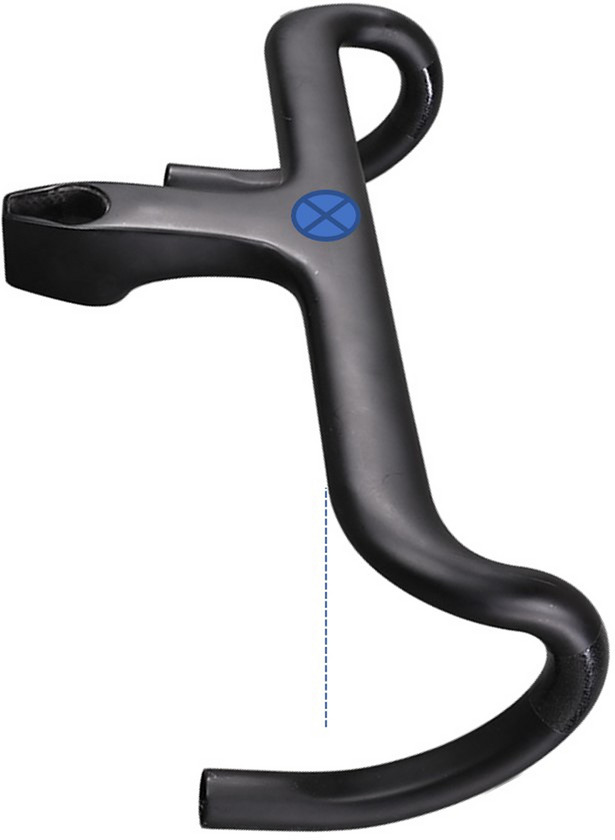
Proposed vertical measurement for integrated handlebars

**Fig. 8:**
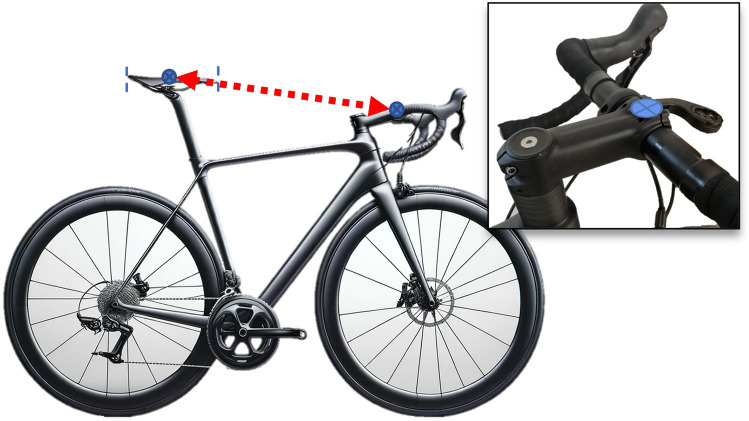
Diagonal distance between handlebar and saddle

1. Bicycle set-up dimensions
**Statement 1.1** Bicycle or cycle ergometer It must be described if the bicycle measurements were performed on the participant’s bicycle or a cycle ergometer If the measurements were performed on a cycle ergometer, one must communicate the level of adjustment possible on the cycle ergometer, which components have been adjusted, and if the cycle ergometer allows continuous or discrete adjustments (Fig. 2), including the fractional adjustments. It is recommended to provide information about how different the cycle-ergometer is to the participant’s bicycle (saddle, crank length, pedals, etc.)
**Statement 1.2** Tools for measurement of bicycle dimensions The instrument or methodology used for the measurement of bicycle dimensions should be described as detailed as possible. It is recommended to include the measurement error of the instrument/methodology. If it is a manual measurement, which means it involves any variable that requires the manipulation and intervention of a tool (measuring tape, caliper, etc.) by the researcher for its measurement, then at least three measurements should be performed and report the mean
**Statement 1.3** Saddle height The saddle height is defined as the distance between the highest point of contact on the saddle by the ischial tuberosities to the pedal spindle. This distance should be measured initially determining the shortest distance between the saddle (as shown in Fig. 3) to the centre of the bottom bracket, then adding the crank arm length to obtain the true saddle height. If determining the location of the ischial tuberosities on the saddle is not possible, the middle point between the larger width of the saddle should be used. If saddle size does not allow to determine this point, the centre of the saddle should be an option. The authors must indicate precisely the locations used for the calculation of the height of the saddle
**Statement 1.4** Saddle setback The saddle setback is regulated by the UCI as the horizontal distance between the saddle nose to the bottom bracket centre. It is recommended to include the UCI horizontal distance in studies and additionally to unify the criteria with the previous statements, and to avoid the effects of different crank lengths and saddle dimensions, it is proposed to include in future investigations the setback information as the horizontal distance between the top point of contact on the saddle by the ischial tuberosities and the pedal spindle. This distance should be measured from the saddle (as shown in Fig. 4) to the centre of the bottom bracket, then add the crank arm length to obtain the true saddle setback. If determining the location of the ischial tuberosities on the saddle is not possible, the middle point between the larger width of the saddle should be used. If saddle size does not allow to determine this point, the centre of the saddle should be an option. The authors must precisely indicate the saddle location
**Statement 1.5** Effective seat tube angle Effective seat tube angle is the angle between a horizontal line running through the centre of the bottom bracket and the line from the bottom bracket to the point of saddle measurement (the ischial tuberosities or the widest point of the saddle, or the centre of the saddle, which must be indicated precisely by the authors) (Fig. 5) The authors must indicate precisely the locations used for the calculation of the centre of the saddle (widest part, ischial tuberosities or centre of the saddle)
**Statement 1.6** Crank length Crank length is the distance between the centre of the bottom bracket and the centre of the pedal axis Note: It must be reported if two different crank lengths are used bilaterally
**Statement 1.7** Vertical difference between handlebars and saddle height The vertical difference between the handlebar and saddle height must be determined as the vertical distance between the top point of contact on the saddle for the ischial tuberosities and the centre of the handlebar clamping point (Fig. 6). If determining the location of the ischial tuberosities on the saddle is not possible, the middle point between the larger width of the saddle should be used. If saddle size does not allow to determine this point, the centre of the saddle should be an option. If the cyclist has integrated handlebars, the handlebar point should be measured as the central intersection between the handlebar and the integrated stem (Fig. 7) The authors must indicate precisely the locations used for the calculation of the centre of the saddle (widest part, ischial tuberosities or centre of the saddle)
**Statement 1.8** Diagonal distance between handlebar and saddle The diagonal distance between the handlebar and saddle measurement point is the distance between the top point of contact on the saddle for the ischial tuberosities (or widest point, or centre of the saddle) and the centre of the handlebars clamping point (Fig. 8). If determining the location of the ischial tuberosities on the saddle is not possible, the middle point between the larger width of the saddle should be used. If saddle size does not allow to determine this point, the centre of the saddle should be an option. If the cyclist has integrated handlebars, the handlebar point should be measured as the central intersection between the handlebar and the integrated stem (Fig. 7) The authors must indicate precisely the locations used for the calculation of the centre of the saddle (ischial tuberosities, widest part or centre of the saddle)

**Table 3:** Statements that obtained a consensus for kinematic analysis after the Delphi procedure

**Fig. 9:**
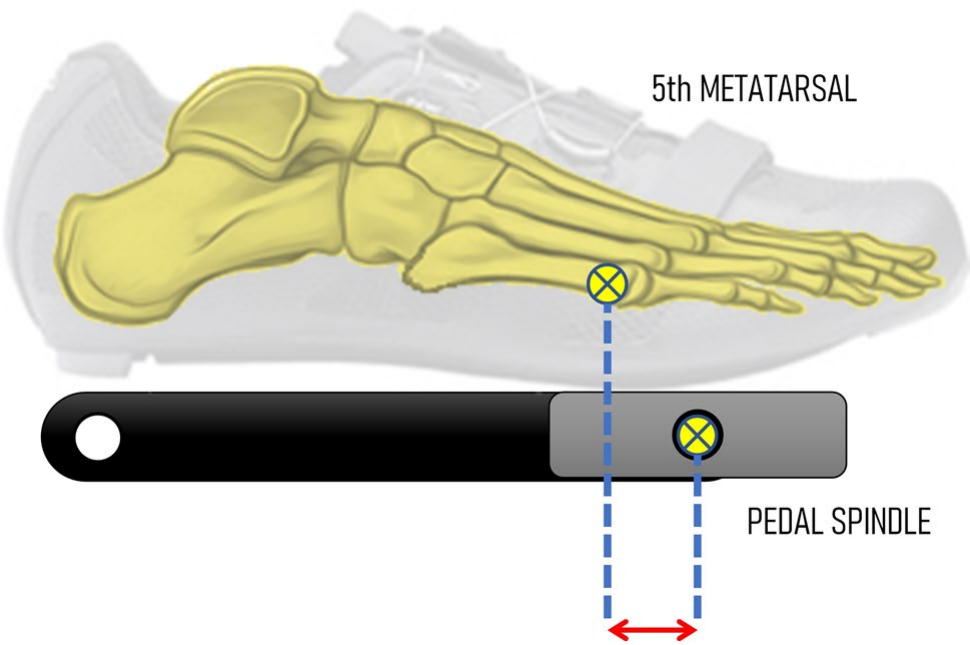
Foot pedal position (red arrow)

2. Kinematic methodology
**Statement 2.1** Conditions Details of exercise testing should be provided, including duration of assessment, intensity, cadence, and rating of perceived exertion
**Statement 2.2** Dynamic assessment Dynamic assessment is encouraged as it has been demonstrated to better reflect the dynamics of posture on the bike compared to static assessments. Assessment should be performed after the cyclist has properly warmed up and pedalled for at least 2 min at the intended intensity and cadence to enable stability in movement patterns. For high-intensity exercises, it is suggested at least 30 s for data acquisition, depending on the participant’s capacity and study design. The number of cycles obtained and analysed should be reported along with how data were summarised (e.g. mean of ten cycles)
**Statement 2.3.** Kinematic 2D versus 3D Whenever possible, 3D kinematic measurements should be obtained instead of 2D. However, if 3D measurements are not available, 2D kinematic data can be gathered stating the limitations related with difficulties in determining joint centres and parallax errors
**Statement 2.4** Normalisation by the individual’s standing posture for bicycle fitting For assessment of movement on the bike, it is recommended to provide the joint angles during cycling and consider that using the individual's standing posture as an offset may benefit the kinematics analysis. This is applicable to individuals with no anatomical or neurological impairment. For this normalisation, static trials should be obtained with the individual standing in anatomical position This normalisation is based in the following calculation: Normalised joint angle = raw joint angle – angle at standing posture More research about this normalisation is necessary before a higher encouragement of its use
**Statement 2.5** Methodological aspects for kinematic measurement and analysis In general, kinematic technologies should be detailed, including but not limited to data recording method (e.g. optoelectronic, video footage, inertial measurement unit [IMU], etc.), sampling frequency (Hz), level of accuracy (i.e. measurement error and sensitivity), reproducibility (intra and inter-sessions), software, data processing, filters used, and definition of the variables obtained. It should be stated the experience of the assessor using the technology and if all the measurements were performed by the same evaluator or a different evaluator Moreover, some methodological aspects must be considered for 2D kinematic and 3D measurements and analysis: Camera position: distance of the camera to the movement plane. Calibration procedures. In the case of 3D kinematics, the number and type of cameras should be provided Calibration: calibration procedures and methods to correct the camera's optical distortion for 2D measurements should be informed if utilised. For 3D kinematics, calibration procedure and motion capture volume available should be informed Kinematic model: type of markers (active, passive), markers size, number and position of markers, and the definition of the angles must be provided. It is encouraged to provide a figure illustrating marker locations and angles and/or segment definitions. For 3D kinematics, a preferred rotational sequence, joint centre determination protocol, and convention of angle polarity should also be provided Recording process: recording time/number of pedalling cycles must be provided Data analysis: software, type of tracking, gap filling, filtering, recording time/number of pedalling cycles and determination of crank/pedal cycle Normalisation: it is desirable to normalise data using the crank angular position (0°–360°) rather than the time when evaluating and comparing between several repetitions to create a mean envelope of any variable
**Statement 2.6** Full-body position during the bicycle fitting assessment Due to the interrelationship between body segments, kinematics should not be limited to the analysis of a single joint of the lower limbs. Although it remains a suggestion, at least the kinematics of the ankle, knee, and hip joints are recommended for reporting Even though the focus may be on assessing the lower limbs, overall body position on the bicycle, including the position of the head, trunk, and hands on the handlebars (e.g. upright position, hand on the grip or dropped posture) should be reported
**Statement 2.7** Report of knee flexion at the bottom of the revolution Knee flexion angle at the bottom of the pedal revolution is often the primary outcome used to determine saddle height. Multiple methods may be used to obtain this outcome, thus producing systematically different results. Therefore, the knee flexion angle method utilised, and numerical outcomes from this angle should be reported. If another method, different than 6 o'clock method, is used, it would be appreciated to include the crank angle at which the knee flexion angle was obtained
**Statement 2.8** Foot pedal position It is recommended to report the foot position on the pedal as the horizontal distance between the first or the fifth metatarsophalangeal joint centre and the pedal spindle when the pedal is parallel with the horizontal axis (Fig. 9)
**Statement 2.9** Sample characterisation When including information about the sample characteristics, it is recommended to report the total number of participants, sex, age, height, body mass, body mass index, weekly training frequency, weekly volume of training (km or hours), years of experience, level, leg preference, etc. When possible/relevant, also report physiological outcomes that may help to understand the sample training level, which includes aerobic capacity or performance in W or W/kg or ml/kg/min, FTP (W or W/kg or ml/kg/min) or similar

## Discussion

This research aimed to obtain a consensus about best practices for measuring and reporting bicycle set-up dimensions and collecting cycling kinematic data. Four core members and fourteen experts agreed on eight statements regarding bicycle measurements and nine statements regarding cycling kinematic assessment. The statements are presented to assist a range of users with differing access to equipment and technology. Best practice guidelines in bicycle measurement and kinematic data analysis can help improve research in the topic and the practical assessment of bicycle fitting. We are confident that further investigation will improve the current proposal and enhance the overall quality of cycling science. We intentionally omitted variables such as apparel, performance, environmental conditions and evaluator experience to maintain methodological clarity and consistency in the present study. We acknowledge the importance of these factors and suggest that future research explores their interactions and impacts in more detail. This approach aims to provide a solid foundation for standardising methods, which can be built upon in subsequent studies to address specific performance-related questions.

The consensus regarding bicycle set-up dimensions and cycling kinematic methodology is closely aligned with existing research, but there are ongoing gaps in the literature. Therefore, it is recommended that the present consensus be considered a guide, not hermetic, with scientific evidence for all its statements. Some statements may have a considerable amount of scientific research behind them, e.g., statement 2.3 regarding the differences between 2 and 3D kinematic measurements [27, 28] or statement 2.6 about the interrelationship between body segments during cycling [29, 30]. A recent study supporting statement 2.6 showed that increased pelvic tilt results in higher hip flexion and a more dorsiflexed ankle angle [30]. However, there are statements for which more investigation is needed (e.g. statement 2.6 ‘Normalisation by the individual’s standing posture for bicycle fitting’). Therefore, this consensus will need ongoing review and modification based on future evidence.

The concluding statements (Tables 2 and 3) are not intended to end the discussion and analysis around bicycle set-up dimensions and cycling kinematics but to act as a starting point for measurement, reporting and communicating. For example, in statement 1.3, aimed at determining saddle height, it is important to consider that while the point of contact on the saddle for the ischial tuberosities could be an option as a reference point, the pelvis can have variability in its antero-posterior position on the saddle. Another option could be assuming the centre of the saddle as a reference. However, this option can also be affected by the geometry of the saddle [31]. Therefore, it is possible that even after standardising the protocol for measuring saddle position, there are still questions about which method can provide the most reliable result. To mitigate these limitations, it was decided to provide multiple options and include clear and justified explanations for replication.

Two statements regarding kinematic data measurement caused great controversy among panellists. These involved the statement around ‘angle definitions’ (for which no consensus was achieved) and statement 2.4 ‘Normalisation by the individual’s standing posture for bicycle fitting’. Concerning ‘angle definitions,’ while the International Society of Biomechanics (ISB) makes recommendations for 3D analysis [32], there is great diversity in the definition of 2D angles for the spine and trunk [7, 33, 34]. These findings and the failure of our expert panel to reach a consensus further demonstrate the complexity of these measures, which seem to result from diverse approaches regarding the number of segments and reference points and suggest the need for future methodological studies. Therefore, carefully describing the methodology for determining 2D angles is recommended. Considering statement 2.4, a previous study using 2D video-based analyses observed a mean bias of ~ 11° in knee flexion comparing absolute angles and angles when normalisation by the individual’s standing posture was performed [35]. However, literature on this topic is scarce, and most studies only provide absolute angles. For this reason, the statement suggests that future research is needed to provide references for both absolute and normalised angles.

This Delphi process yielded expert consensus on performing bicycle measurements and kinematic analysis for cycling research and testing. This consensus is an important step towards the standardisation of measures to optimise the bicycle fitting process for recreation, rehabilitation and competition.

The review highlighted key areas where additional research is needed and suggested important next steps:

1. Generally accepted recommendations for reporting the whole body’s cycling kinematics (e.g. joint coordinate systems) in 2D and 3D are necessary before the field can move forward.
2. Research is required to determine if and how normalising kinematic variables should be performed; factors to consider are 2D and 3D data, joints and rider type (competitive, recreational or rehabilitation), as the developed statements are not specific to any rider type. Furthermore, it is important to understand that the statements may require adjustment to cater to populations with disabilities or injuries [36, 37], and future consensus could focus on such populations.
3. Future research and consensus statement development must consider different measurement conditions, e.g. 2D versus 3D, static versus dynamic and optical (camera) versus inertial measurement unit (IMU), to provide recommendations on body position during cycling.
4. Future consensus statements should consider measuring and reporting additional aspects of cycling biomechanics, e.g. how forces and moments are collected, how these data are processed, how joint kinetics are reported (joint reaction, contact forces, or joint moments and power) and how muscle activation (electromyography, simulation-based approaches) are computed and reported.

Despite the limitations, we argue that this consensus will benefit the field of study and improve the quality of bicycle fitting and cycling research. Moreover, the statements can help reviewers during the peer review process of cycling studies and guide authors to adhere to best practices in their reports. In this sense, two appendices (Appendix 1 and Appendix 2) are provided to ensure that all essential data were considered and included in the report. Core and panel members suggest using the present consensus to guide research studies.

## Conclusions

We present a consensus of eight statements for measuring and reporting bicycle set-up dimensions and nine statements for cycling kinematics data collection for a wide array of researchers and cycling related practitioners. We encourage scientists and professionals to apply these statements in the field, aiming to improve the reproducibility, standardisation and interpretation of bicycle fitting assessments and cyclist testing protocols.

## Electronic supplementary material

Below is the link to the electronic supplementary material.

Supplementary file1 (DOCX 1531 KB)

## Appendix 1

Checklist about the bicycle set-up dimensions consensus to ensure that all the important information was considered and included in a report or a scientific paper.

The appendices provide a structured set of recommendations derived from the consensus reached in this article. To use them effectively, we recommend the following:

- **Comprehensive review:** use the appendices as a checklist to review all aspects analysed and agreed upon in your study of cycling biomechanics.
- **Marking and description:** mark the corresponding option in the brackets provided and then describe that option in detail in your study, ensuring coherence and clarity in applying the recommendations.

**Table:**

Item	Description
1. Bicycle set-up dimensions	- Indicate the adjusted components: [] Saddle height [] Saddle setback [] Crank length [] Vertical Difference between Handlebars and Saddle Height [] Diagonal Distance between Handlebar and Saddle [] Others: __________________ - Indicate whether the measurements were performed on the participant's bicycle or a cycle ergometer [] participant's bicycle [] cycle ergometer - When using a cycle ergometer, add information: *Adjustments:* [] Adjustments continuous [] Adjustments discrete *Information:* [] Model and version, Brand company, city, country [] Software to control [] Others: __________________
2. Tools for measurement of bicycle set-up dimensions	- Indicate the tools employed to measure the bicycle dimensions: [] Videography [] Measuring tape [] Goniometer [] Laser [] Others: __________________ - Measurement information: [] Measurement error [] If manual measurement: conduct at least **three** measurements and report the mean
3. Saddle height	- Define saddle height and describe the measurement procedure [] Definition - Locations used for the calculation of saddle height *From:* [] Centre of the bottom bracket *Add:* [] Crank arm length *To:* [] Ischial tuberosities [] Middle point between the larger width of the saddle [] Centre of the saddle
4. Saddle setback	- Define saddle setback and describe the measurement procedure [] Definition [] UCI rules definition - Locations used for the calculation of saddle setback *From:* [] Centre of the bottom bracket *Add or not:* [] Crank arm length *To* [] Ischial tuberosities [] Middle point between the larger width of the saddle [] Centre of the saddle
5. Effective seat tube angle	- Define effective seat tube angle and describe the measurement procedure [] Definition - Locations used for the calculation of the effective seat tube angle *From:* [] Horizontal line running through the centre of the bottom bracket *To:* [] Line from the bottom bracket to the point of the saddle [] Ischial tuberosities [] Middle point between the larger width of the saddle [] Centre of the saddle
6. Crank length	- Define crank length [] Definition - Are two different lengths used bilaterally? [] Yes [] No
7. Vertical difference between handlebars and saddle height	- Define the vertical difference between handlebars and saddle height [] Definition - Locations used to calculate the vertical difference between handlebars and saddle height *Saddle height point selected:* [] Ischial tuberosities [] Middle point between the larger width of the saddle [] Centre of the saddle *Handlebar point selected:* [] Clamping point [] If integrated handlebar: central intersection between the handlebar and the integrated stem
8. Diagonal distance between handlebar and saddle	Define the diagonal distance between the handlebars and the saddle [] Definition - Locations used to calculate the diagonal distance between handlebars and saddle *Saddle height point selected:* [] Ischial tuberosities [] Middle point between the larger width of the saddle [] Centre of the saddle *Handlebar point selected:* [] Clamping point [] If integrated handlebar: central intersection between the handlebar and the integrated stem

## Appendix 2

Checklist about the kinematics methodology consensus to ensure that all the necessary information is considered and included in a report or a scientific paper.

The appendices provide a structured set of recommendations derived from the consensus reached in this article. To use them effectively, we recommend the following:

- **Comprehensive review:** Use the appendices as a checklist to review all aspects analysed and agreed upon in your study of cycling biomechanics.
- **Marking and description:** Mark the corresponding option in the brackets provided and then describe that option in detail in your study, ensuring coherence and clarity in applying the recommendations.

**Table:**

Item	Description
1. Sample	- Indicate the **sample characteristics**: [] number of participants [] Sex [] Age [] Height [] Body mass [] Body mass index [] Weekly training frequency [] Weekly volume of training (km or hours) [] Years of experience [] Level [] Leg preference [] - Indicate when possible/relevant **physiological outcomes**: [] Aerobic capacity performance (W or W/kg or ml/kg/min) [] FTP (W or W/kg or ml/kg/min) [] Others: __________________
2. Study approach, protocol, and recording conditions	- Indicate the **type of approach** carried out: [] Dynamic 2D assessment [] Dynamic 3D assessment [] Static assessment [] Anthropometric assessment - Indicate the **protocol** carried out [] Warm up protocol [] Recording time lapses - For submaximal intensities: at least 2 min at the intended intensity and cadence to enable stability in movement patterns - For high-intensity exercises: at least 30 s at the intended intensity - Exercise testing conditions: [] Duration [] Intensity [] Cadence [] Rating of perceived exertion [] Others: __________________ - Indicate the **experience of the assesso**r who carried out the study: [] Years of experience [] Indicate if all the measurements were performed by the same evaluator or a different evaluator
3. Collecting data and methodological aspects	If dynamic assessment was performed, indicate the following aspects: - Indicate **kinematic technology** employed: [] Recording method (e.g., optoelectronic, video footage, inertial measurement unit (IMU), etc.) [] Sampling frequency (Hz) [] Level of accuracy (i.e., measurement error and sensitivity if possible) [] Reproducibility (intra and inter-sessions if possible) [] Software (Model and version, Brand company, city, country) - Indicate **methodological aspects** employed: [] Number and type of cameras (if 3D analysis) [] Calibrate procedures for 2D and 3D approaches [] Kinematic model [] Number and type (passive or active) of markers [] Marker size [] Anatomical position [] Angle definition [] Recommended to include a figure with location, angle/segment definitions [] Indicate joint centre determination (3D) - Indicate **data processing** employed: [] If your data has been normalised to the **individual's standing posture**: [] Yes, indicate how it was normalised [] No [] Number of cycles obtained and analysed (e.g., mean of ten cycles) [] Data processing [] Type of tracking [] Gap filling, filtering [] Filters used [] Determination of crank/pedal cycle [] Definition of the variables [] Knee flexion angle method (preferably 6 o'clock method; indicate if another method is used) [] Foot pedal position

## Declarations

### Data availability

The data that support the consensus are available on request from the corresponding author.
