# Supplementary material for: Bicycle Set-Up Dimensions and Cycling Kinematics: A Consensus Statement Using Delphi Methodology
Source: Sports Med. 2024 Sep 20;54(11):2701–15. doi: 10.1007/s40279-024-02100-6 (PMC11561003; doi:10.1007/s40279-024-02100-6)
Supplement: Supplementary file 1 — Supplementary file1 (DOCX 1531 KB) [file 40279_2024_2100_MOESM1_ESM.docx]

| **Members** |  |
| --- | --- |
| **Round** | First |
| **Date** | DD/MM/YYYY |

**INSTRUCTIONS**

Thank you for agreeing to participate in this project. Below we provide some instruction for the next steps. We hope the process can run as smoothly as possible, so if you have any questions, please do not hesitate to contact [delphicycling@gmail.com](mailto:delphicycling@gmail.com).The Delphi method consists in an expert consensus which is undertaken surveying expert opinion and reaching a group response (Steurer 2011). You have the right to co-author the final manuscript and congress communication if you fully meet the eligibility criteria. For that, you are required to complete this document in 15 days and approve the final version of the manuscript. The two core members that lead the project will be the first and last author, and the rest of the authors will appear following the alphabetic order of their surname.

Instructions:

- Rate each item according to a five-point Likert Scale. **You are rating the “statement”**, that it is which we are trying to obtain consensus. The “research background” is just the information that we are providing to support our statement, but it will not appear in the future article as information that we have agreed.
- In the “comments box” you can provide input in any of the items included in the instrument. You can also suggest possible rephrasing of questions and you can highlight anything that may have been missed from the initial list of items. We encourage you to support your answers with scientific arguments based on published studies. When it was not possible to obtain references to support decisions, we suggest explaining your positions considering your practical experience.
- At the end of the document, you will find the references used for each item.
- To balance opinions from different research groups, members of the same research group must fill the document in conjunction, on behalf of all, demonstrating group consensus.
- **Please bear in mind that you should not evaluate the items purely based on experience or personal preferences.** The study has with two main aims:
  - To state a consensus about how to perform bike measurements in male and female cyclists.
  - To propose a checklist of essential aspects to be considered in the kinematic assessment of cyclist’s posture adjustment.

**We advocate an empathic attitude that allows a consensus to be reached. Thank you in advance for collaborating in this project!**

| **1.BICYCLE MEASUREMENTS SECTION** |
| --- |

**This section intends to obtain consensus on how to perform bicycle measurements.**

| **ITEM 1.1 BICYCLE OR CYCLE ERGOMETER** |
| --- |
| **Research background (not for consensus):** The biomechanics assessment of cyclists can be performed using cyclists’ own bicycle or a type of cycle ergometer. The equipment used should be reported in the manuscript and related documents, because different bicycles will allow for various fitting (saddle height, saddle angle, handlebar position, etc.) as well as the influence of equipment familiarization. In the case of a cycle ergometer, it should be reported if the equipment allows a full configuration or if the adjustments are made discreetly (e.g., the guides that mark the changes in saddle height are every 1.5 cm). |
| **Statement (for consensus) 1.1.1:**  **It must be described if the bicycle measurements were performed in the participant own bicycle or in a cycle ergometer.** |
| **Evaluation of the item 1.1.1:**  5.Strongly agree  4.Moderately agree  3.Neutral  2.Moderately disagree  1.Strongly disagree |
| **Statement (for consensus) 1.1.2:**  **It the measurements were performed in a cycle ergometer, it must be informed if cycle ergometer allows continuous or discrete adjustments (it must be informed about the minimum distance between possible adjustments).** |
| **Evaluation of the item 1.1.2:**  5.Strongly agree  4.Moderately agree  3.Neutral  2.Moderately disagree  1.Strongly disagree |
| **Panel member comments:** |

| **ITEM 1.2 TOOLS FOR MEASUREMENT OF BICYCLE DIMENSIONS** |
| --- |
| **Research background:** Measurements of bicycle dimensions can be performed in different ways: manually and directly, for example using an anthropometric tape (Ferrer-Roca *et al.* 2014), with calculations from the metrics guides and marks of the bicycle/cycle-ergometer (Gatti *et al.* 2021), or inferred based on image technology. Due to the possible systematic errors for each measurement, this information must be stated in the manuscript and related documents. |
| **Statement:**  **Instrument or methodology used for bicycle measurements must be described as detailed as possible.** |
| **Evaluation of the item:**  5.Strongly agree  4.Moderately agree  3.Neutral  2.Moderately disagree  1.Strongly disagree |
| **Panel member comments:** |

| **ITEM 1.3 SADDLE HEIGHT** |
| --- |
| **Research background:** To define the saddle height, there are two points of measurement, the upper and the lower point.  In regards to the upper point, it can be defined as the top of the saddle aligned with the top of the seat tube (Verma *et al.* 2016, Gatti *et al.* 2021, Millour *et al.* 2021) or the centre of the saddle. The centre of the saddle can be affected by saddles with different geometries, but the alignment with the tube can be influenced by bikes with different geometries. In addition, this method may not very accuracy if the centre of the saddle is not aligned with the seat tube. For this reason, we have decided to opt for the centre of the saddle. |
| **Statement:**  **The saddle height is defined by the distance between the top of the centre of the saddle and the bottom bracket adding the crank arm.**  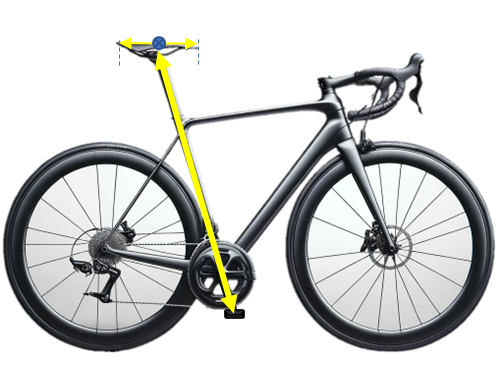  **Figure 1.** Proposed saddle height measurement. |
| **Evaluation of the item:**  5.Strongly agree  4.Moderately agree  3.Neutral  2.Moderately disagree  1.Strongly disagree |
| **Panel member comments:** |

| **ITEM 1.4 SADDLE SETBACK** |
| --- |
| **Research background:** Saddle setback is commonly defined as the horizontal distance between the centre of the saddle and the bottom bracket, which also satisfies an UCI rule (1.3.013) determining that “The peak of the saddle shall be a minimum of 5 cm to the rear of a vertical plane passing through the bottom bracket spindle”. The different saddle geometries limit this definition. Considering this idea, Menard et al. (2020) evaluated saddle setback position as a distance between centre of pressure on the saddle and bottom bracket. Moreover, Gatti et al. (2021) considered saddle setback as the horizontal distance from the bottom bracket to the centre of the saddle clamp. To perform the measurement from centre of the saddle until the pedal axis also presents the advantage that is considering the crank length. |
| **Statement:**  **The saddle setback is the horizontal distance between the top of the centre of the saddle and the pedal axis with the crank arm aligned horizontally (at 3 o’clock).**  **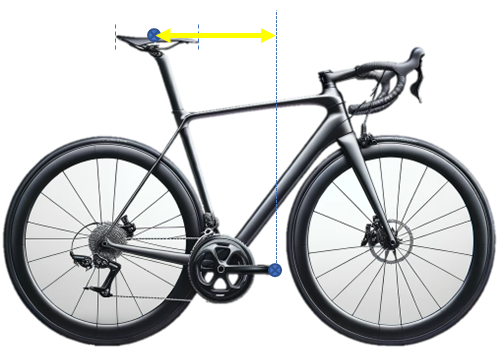**  **Figure 2.** Proposed saddle setback measurement. |
| **Evaluation of the item:**  5.Strongly agree  4.Moderately agree  3.Neutral  2.Moderately disagree  1.Strongly disagree |
| **Panel member comments:** |

| **ITEM 1.5 SADDLE TUBE ANGLE** |
| --- |
| **Research background:** We understand that this variable is well defined since it is quite geometric and provided by bicycle manufacturers. |
| **Statement:**  **The saddle tube angle is defined as the angle between the seat tube and a horizontal line running through the bottom bracket.** |
| **Evaluation of the item:**  5.Strongly agree  4.Moderately agree  3.Neutral  2.Moderately disagree  1.Strongly disagree |
| **Panel member comments:** |

| **ITEM 1.6 CRANK LENGHT** |
| --- |
| **Research background:** We understand that this variable is well defined since it is quite geometric and provided by bicycle manufacturers. |
| **Statement:**  **Crank length is the distance between the bottom bracket and pedal axis, and it is commonly indicated by the manufacturer in millimetres.** |
| **Evaluation of the item:**  5.Strongly agree  4.Moderately agree  3.Neutral  2.Moderately disagree  1.Strongly disagree |
| **Panel member comments:** |

| **ITEM 1.7 VERTICAL DIFFERENCE BETWEEN HANDLEBARS AND SADDLE HEIGHT** |
| --- |
| **Research background:** As for the saddle height, handlebars position requires measurements in two points: the saddle and the handlebar.  For the saddle point, we understand that the same point used for determination of saddle height can be used: the upper point of the centre of the saddle.  The centre of the handlebars has been used as the handlebars point (Korff *et al.* 2011, Ferrer-Roca *et al.* 2014). |
| **Statement:**  **Vertical difference between handlebar and saddle height must be determined as the difference in the vertical distance between the top of the centre of the saddle and the centre of the handlebar.**  **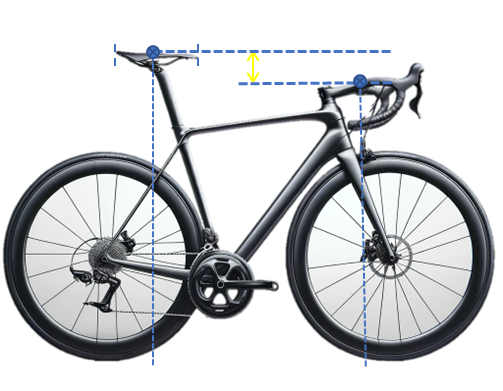**  **Figure 3.** Proposed vertical difference between handlebar and saddle height measurement. |
| **Evaluation of the item:**  5.Strongly agree  4.Moderately agree  3.Neutral  2.Moderately disagree  1.Strongly disagree |
| **Panel member comments:** |

| **ITEM 1.8 HORIZONTAL DISTANCE BETWEEN HANDLEBAR AND SADDLE** |
| --- |
| **Research background:** Previous studies defined this measurement as the horizontal distance between the centre of the saddle and the centre of the handlebar (Korff *et al.* 2011, Ferrer-Roca *et al.* 2014), or considering the front tip of the saddle (Verma *et al.* 2016).  We think that in this case, it is important to consider the hand point of support, the handlebar grips, for the effect of different handlebar geometries.  Our proposal is to measure the shortest distance (see Figure 3) because it will consider in a better way the saddle characteristics.  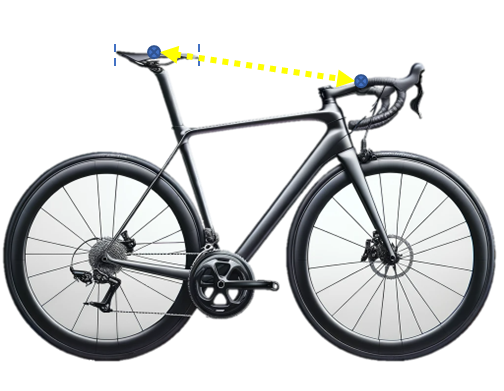  **Figure 4.** Difference between diagonal and horizontal distance. |
| **Statement:**  **Horizontal distance between handlebar and saddle is the diagonal distance between the top of the centre of the saddle and the handlebar grips.** |
| **Evaluation of the item:**  5.Strongly agree  4.Moderately agree  3.Neutral  2.Moderately disagree  1.Strongly disagree |
| **Panel member comments:** |

| **2.KINEMATIC METHODOLOGY SECTION** |
| --- |

**This section has the aim to achieve a consensus about recommendations to perform kinematic analysis.**

| **ITEM 2.1 CONDITIONS** |
| --- |
| **Research background:** Body position on the bicycle is sensitive to changes in exercise conditions such as exercise workload and/or intensity (Bini et al., 2019), cadence (Bini, Rossato, et al., 2010), and fatigue state (Bini, Diefenthaeler, et al., 2010). |
| **Statement:**  **Details of exercise conditions must be provided, including duration of assessment, workload, cadence, and rate of perceived exertion.** |
| **Evaluation of the item:**  5.Strongly agree  4.Moderately agree  3.Neutral  2.Moderately disagree  1.Strongly disagree |
| **Panel member comments:** |

| **ITEM 2.2 DYNAMIC ASSESSMENT** |
| --- |
| **Research background:** Measurements of angles taken statically and dynamically are often different because movement creates angular momentum, which is not observed in static poses (Holliday *et al.* 2017, Bini and Hume 2018, Millour *et al.* 2019). Differences of 5 ±1°, 8 ±2°, and 9 ±2° were observed between static and dynamic methods at 6 o'clock in the hip, knee, and ankle angle, respectively (Bini and Hume 2018). Although, we think that it is important to consider a dynamic assessment, it is difficult to propose a minimum time measurement because it can be different depending of the objective of the study or the instrument used. |
| **Statement:**  **Dynamic assessment is encouraged as it has demonstrated that results of measuring static angles are not equivalent to dynamic angles. The minimum measurement time should be chosen according to the sampling frequency of the instrument used and the objective of the study.** |
| **Evaluation of the item:**  5.Strongly agree  4.Moderately agree  3.Neutral  2.Moderately disagree  1.Strongly disagree |
| **Panel member comments:** |

| **ITEM 2.3 KINEMATIC METHOD USED** |
| --- |
| **Research background:**  Different technologies can be used to determine body position on the bicycle, some of them are validated and other not. In this sense, new technologies involving inertial measurement units and marker-less methods have been introduced to assess cyclists' movement and body position on the bicycle (Grigg *et al.* 2018, Bini and Hume 2020, Bini *et al.* 2021). It is important to note that commercially available devices need studies of validity and reliability analysis before its kinematic data are used to help determining the position of the cyclist (FitzGibbon *et al.* 2017). |
| **Statement:**  **Kinematic technologies should be detailed including but not limited to sampling frequency, accuracy, measurement error, and resolution. Moreover, it is encouraged to support the use of validated kinematic technologies.** |
| **Evaluation of the item:**  5.Strongly agree  4.Moderately agree  3.Neutral  2.Moderately disagree  1.Strongly disagree |
| **Panel member comments:** |

| **ITEM 2.4 KINEMATIC 2D vs. 3D** |
| --- |
| **Research background:** Even though cyclists move their segments largely in the sagittal plane, the transverse plane and mediolateral movements are also part of the pedalling motion (Carpes *et al.* 2009, Bini *et al.* 2016). Furthermore, the methods to determine joint angles are different when data is obtained using two- or three-dimensional measurements (for example, 3D measurements determine the joint centre of rotation while 2D consider fix references). These lead to 3 to 10° differences between three- and two-dimensional angles (Umberger and Martin 2001, Fonda *et al.* 2014). This element adds an important confounder to the use of simple methods such as sagittal plane video recording in bicycle fitting. Therefore, when using two-dimensional derived joint angles, it is assumed that limitations will exist in relation to the gold-standard assessment (i.e., three-dimensional). |
| **Statement:**  **Whenever possible, 3D kinematic analysis must be performed instead of 2D. However, if 3D analysis is not available, kinematic 2D can be performed but always stating the limitations considering the determination of joint angle and the influence of out of plane movements.** |
| **Evaluation of the item:**  5.Strongly agree  4.Moderately agree  3.Neutral  2.Moderately disagree  1.Strongly disagree |
| **Panel member comments:** |

| **ITEM 2.5 NORMALIZATION BY THE INDIVIDUAL’S STANDING POSTURE** |
| --- |
| **Research background:** When conducting three-dimensional analysis a reference posture taken from the participant at standing posture is considered in the calculations (Nielsen and Daugaard 2008, Jones *et al.* 2009, Maykut *et al.* 2015). For two-dimensional analysis in cycling, this principle has been initially explored and revealed average bias of ~11° [8-13° confidence interval for differences] in knee flexion angles when a standing posture was taken into account (Priego Quesada *et al.* 2016). Despite benefits of using both 2D or 3D approaches to assess cycling posture, evidence in this topic is limited, particularly when proposing articular ranges of motion in bicycle fitting or when predictive equations are developed to determine bicycle fitting based on joint angles. Moreover, most studies did not state clearly if they normalized or not angles considering a reference from a standing posture. |
| **Statement:**  **Joint angles at the individual’s standing posture must be considered as an offset to normalize angles during cycling. Barefoot upright keeping the knees completely extended must be the standing posture performed. This normalization is based in the following calculation:**  **Normalized angle = non-normalized – angle at standing posture** |
| **Evaluation of the item:**  5.Strongly agree  4.Moderately agree  3.Neutral  2.Moderately disagree  1.Strongly disagree |
| **Panel member comments:** |

| **ITEM 2.6 METHODOLOGIAL ASPECTS FOR 2D ANALYSIS** |
| --- |
| **Research background:** We have included some of the most important aspects to take into account for a reliable 2D analysis (Page *et al.* 2008) |
| **Statement:**  **Some methodological aspects must be considering for kinematic 2D analysis and must be informed:**  **- Camera position: Camera plane must be parallel to the movement plane.**  **- Frame calibration: Calibration procedure should be performed to correct angles deviations and camera’s optical distortion.**  **- Kinematic model: Number and position of markers, and the definition of the angles must be provided. It is encouraged to provide a figure illustrating marker model location.**  **- Recording features: Sampling rate (Hz) and recording time/number of pedalling cycles must also be provided.** |
| **Evaluation of the item:**  5.Strongly agree  4.Moderately agree  3.Neutral  2.Moderately disagree  1.Strongly disagree |
| **Panel member comments:** |

| **ITEM 2.7 METHODOLOGIAL ASPECTS FOR 3D ANALYSIS** |
| --- |
| **Research background:** We have included some of the most important aspects to take into account for 3D analysis. |
| **Statement:**  **Details about kinematic 3D procedures must be provided:**  **- Camera’s system: number and type of cameras, camera resolution and sampling rate**  **- Calibration procedure**  **- Kinematic model: type of markers (active, passive), number of markers, and its anatomical position, reconstruction method for definition of the angles**  **- Data analysis: filtering, gap filling, recording time/number of pedalling cycles**  **It is encouraged to provide a figure illustrating marker model location.** |
| **Evaluation of the item:**  5.Strongly agree  4.Moderately agree  3.Neutral  2.Moderately disagree  1.Strongly disagree |
| **Panel member comments:** |

| **ITEM 2.8 METHODOLOGIAL ASPECTS FOR OTHER KINEMATIC PROCEDURES** |
| --- |
| **Research background:** This item is proposed to guide any other type of kinematics instrument. |
| **Statement:**  **Methodological aspects and the steps of the kinematic procedures must be provided to ensure the reproducibility and the interpretation of the data by future studies. It is recommended to provide the following information:**  **- Procedure and instrumentation.**  **- Methodology for data analysis: sampling rate, filtering, or other signal processing methods.**  **- Software: describe the software used to collect and analyse data despite whether commercial or customized.**  **- Variables: definition of the variables obtained.** |
| **Evaluation of the item:**  5.Strongly agree  4.Moderately agree  3.Neutral  2.Moderately disagree  1.Strongly disagree |
| **Panel member comments:** |

| **ITEM 2.9 NOT ONLY KNEE FLEXION** |
| --- |
| **Research background:** The pedalling movement involves an interaction between the pelvis and the spine due to role of hip extensor muscles such as gluteus maximus and biceps femoris, whose muscle length–tension relationship are strongly influenced by changes in the hip flexion angle during exercise (Too 1994, Jobson *et al.* 2008). For this reason, changes in saddle position can affect trunk behaviour and vice versa (Too 1994, Salai *et al.* 1999, Fonda *et al.* 2011). Moreover, different studies showed how fatigue may alter in a higher intensity or at least at similar level ankle than knee kinematics (Bini *et al.* 2010, Holliday *et al.* 2019). All of these encourages that kinematic data of multiples joints must be provided to better understand the effect of an intervention. |
| **Statement:**  **Due to the interrelationship between body segments, the kinematic must not be limited to the analysis of a single joint. Although it is a suggestion, depending on the objective of the study, at least the kinematics of the ankle, knee, hip, trunk, shoulder and elbow joints are recommended.** |
| **Evaluation of the item:**  5.Strongly agree  4.Moderately agree  3.Neutral  2.Moderately disagree  1.Strongly disagree |
| **Panel member comments:** |

| **3.END OF THE QUESTIONNAIRE** |
| --- |

**If you think that an item should be added, please write state this in the box below. Please, look for a structure like the one shown in the document, provide the background, references, and the status to be agreed.**

| **COMMENTS** |
| --- |
|  |

**References**

Bailey, M., Maillardet, F., and Messenger, N., 2003. Kinematics of cycling in relation to anterior knee pain and patellar tendinitis. *Journal of sports sciences*, 21 (8), 649–657.

Bini, R. and Hume, P., 2020. Reproducibility of lower limb motion and forces during stationary submaximal pedalling using wearable motion tracking sensors. *Sports Biomechanics*, 0 (0), 1–22.

Bini, R., Serrancolí, G., Santiago, P., and Moura, F., 2021. ASSESSMENT OF A MARKLESS MOTION TRACKING METHOD TO DETERMINE BODY POSITION ON THE BIKE. *ISBS Proceedings Archive*, 39 (1), 37.

Bini, R.R., Dagnese, F., Rocha, E., Silveira, M.C., Carpes, F.P., and Mota, C.B., 2016. Three-dimensional kinematics of competitive and recreational cyclists across different workloads during cycling. *European Journal of Sport Science*, 16 (5), 553–559.

Bini, R.R., Diefenthaeler, F., and Mota, C.B., 2010. Fatigue effects on the coordinative pattern during cycling: Kinetics and kinematics evaluation. *Journal of Electromyography and Kinesiology*, 20 (1), 102–107.

Bini, R.R. and Hume, P., 2018. A comparison of static and dynamic measures of lower limb joint angles in cycling: application to bicycle fitting. *Human Movement*, 17 (1), 36–42.

Bini, R.R., Hume, P.A., and Croft, J., 2014. Cyclists and triathletes have different body positions on the bicycle. *European Journal of Sport Science*, 14 (sup1), S109–S115.

Bini, R.R., Hume, P.A., Lanferdini, F.J., and Vaz, M.A., 2013. Effects of moving forward or backward on the saddle on knee joint forces during cycling. *Physical Therapy in Sport: Official Journal of the Association of Chartered Physiotherapists in Sports Medicine*, 14 (1), 23–27.

Blocken, B., van Druenen, T., Toparlar, Y., and Andrianne, T., 2018. Aerodynamic analysis of different cyclist hill descent positions. *Journal of Wind Engineering and Industrial Aerodynamics*, 181, 27–45.

Carpes, F.P., Dagnese, F., Mota, C.B., and Stefanyshyn, D.J., 2009. Cycling with noncircular chainring system changes the three-dimensional kinematics of the lower limbs. *Sports Biomechanics*, 8 (4), 275–283.

Carpes, F.P., Mota, C.B., and Faria, I.E., 2010. On the bilateral asymmetry during running and cycling - a review considering leg preference. *Physical Therapy in Sport: Official Journal of the Association of Chartered Physiotherapists in Sports Medicine*, 11 (4), 136–142.

Coyle, E.F., Feltner, M.E., Kautz, S.A., Hamilton, M.T., Montain, S.J., Baylor, A.M., Abraham, L.D., and Petrek, G.W., 1991. Physiological and biomechanical factors associated with elite endurance cycling performance. *Medicine and science in sports and exercise*, 23 (1), 93–107.

Crews, R.T., Smith, S.R., Ghazizadeh, R., Yalla, S.V., and Wu, S.C., 2017. Preliminary Evaluation of a Cycling Cleat Designed for Diabetic Foot Ulcers. *Journal of the American Podiatric Medical Association*, 107 (6), 475–482.

Drake, G., 1998. A perfect position. *In*: E. Pavelka, ed. *Bicycling Magazine’s Complete Book of Road Cycling Skills: Your Guide to Riding Faster, Stronger, Longer, and Safer*. USA: Rodale.

Encarnación-Martínez, A., Ferrer-Roca, V., and García-López, J., 2021. Influence of Sex on Current Methods of Adjusting Saddle Height in Indoor Cycling. *Journal of Strength and Conditioning Research*, 35 (2), 519–526.

Ferrer-Roca, V., Bescós, R., Roig, A., Galilea, P., Valero, O., and García-López, J., 2014. Acute effects of small changes in bicycle saddle height on gross efficiency and lower limb kinematics. *Journal of Strength and Conditioning Research*, 28 (3), 784–791.

Ferrer-Roca, V., Roig, A., Galilea, P., and García-López, J., 2012. Influence of saddle height on lower limb kinematics in well-trained cyclists: static vs. Dynamic evaluation in bike fitting. *The Journal of Strength & Conditioning Research*, 26 (11), 3025–3029.

Fintelman, D.M., Sterling, M., Hemida, H., and Li, F.-X., 2014. Optimal cycling time trial position models: Aerodynamics versus power output and metabolic energy. *Journal of Biomechanics*, 47 (8), 1894–1898.

FitzGibbon, S.A., Vicenzino, B., Rauh, M.J., Nichols, J.F., and Sisto, S.A., 2017. Kinematic Measures Of The Knee While Cycling: A Comparison Of Vicon And RetÜL 3d Motion Analysis Systems: 1384 Board# 59 June 1 9: 00 AM-10: 30 AM. *Medicine & Science in Sports & Exercise*, 49 (5S), 377.

Fonda, B., Panjan, A., Markovic, G., and Sarabon, N., 2011. Adjusted saddle position counteracts the modified muscle activation patterns during uphill cycling. *Journal of Electromyography and Kinesiology: Official Journal of the International Society of Electrophysiological Kinesiology*, 21 (5), 854–860.

Fonda, B., Sarabon, N., and Li, F.-X., 2014. Validity and reliability of different kinematics methods used for bike fitting. *Journal of sports sciences*, 32 (10), 940–946.

García-López, J., Díez-Leal, S., Ogueta-Alday, A., Larrazabal, J., and Rodríguez-Marroyo, J.A., 2016. Differences in pedalling technique between road cyclists of different competitive levels. *Journal of Sports Sciences*, 34 (17), 1619–1626.

Gatti, A.A., Keir, P.J., Noseworthy, M.D., Beauchamp, M.K., and Maly, M.R., 2021. Equations to Prescribe Bicycle Saddle Height based on Desired Joint Kinematics and Bicycle Geometry. *European Journal of Sport Science*, 1–10.

Grigg, J., Haakonssen, E., Rathbone, E., Orr, R., and Keogh, J.W., 2018. The validity and intra-tester reliability of markerless motion capture to analyse kinematics of the BMX Supercross gate start. *Sports biomechanics*, 17 (3), 383–401.

Holliday, W., Fisher, J., Theo, R., and Swart, J., 2017. Static versus dynamic kinematics in cyclists: A comparison of goniometer, inclinometer and 3D motion capture. *European Journal of Sport Science*, 17 (9), 1129–1142.

Holliday, W., Theo, R., Fisher, J., and Swart, J., 2019. Cycling: joint kinematics and muscle activity during differing intensities. *Sports Biomechanics*, 0 (0), 1–15.

Jobson, S.A., Nevill, A.M., George, S.R., Jeukendrup, A.E., and Passfield, L., 2008. Influence of body position when considering the ecological validity of laboratory time-trial cycling performance. *Journal of sports sciences*, 26 (12), 1269–1278.

Jones, P.L., Kerwin, D.G., Irwin, G., and Nokes, L.D., 2009. Three Dimensional Analysis of Knee Biomechanics when Landing on Natural Turf and Football Turf. *Journal of Medical and Biological Engineering*, 29 (4), 184–188.

Korff, T., Fletcher, G., Brown, D., and Romer, L.M., 2011. Effect of “Pose” cycling on efficiency and pedaling mechanics. *European Journal of Applied Physiology*, 111 (6), 1177–1186.

Kotler, D.H., Babu, A.N., and Robidoux, G., 2016. Prevention, Evaluation, and Rehabilitation of Cycling-Related Injury. *Current Sports Medicine Reports*, 15 (3), 199–206.

Maykut, J.N., Taylor‐Haas, J.A., Paterno, M.V., DiCesare, C.A., and Ford, K.R., 2015. CONCURRENT VALIDITY AND RELIABILITY OF 2D KINEMATIC ANALYSIS OF FRONTAL PLANE MOTION DURING RUNNING. *International Journal of Sports Physical Therapy*, 10 (2), 136–146.

Menard, M., Domalain, M., Decatoire, A., and Lacouture, P., 2020. Influence of saddle setback on knee joint forces in cycling. *Sports Biomechanics*, 19 (2), 245–257.

Millour, G., Duc, S., Puel, F., and Bertucci, W., 2019. Comparison of static and dynamic methods based on knee kinematics to determine optimal saddle height in cycling. *Acta of Bioengineering and Biomechanics*, 21 (4), 93–99.

Millour, G., Duc, S., Puel, F., and Bertucci, W., 2020. Effect of asymmetric crank arm lengths on performance-related variables in cyclists with an anatomical lower limb length discrepancy. *Sports Engineering*, 23 (1), 14.

Millour, G., Duc, S., Puel, F., and Bertucci, W., 2021. Comparison of two static methods of saddle height adjustment for cyclists of different morphologies. *Sports Biomechanics*, 20 (4), 391–406.

Nielsen, D.B. and Daugaard, M., 2008. Comparison of angular measurements by 2D and 3D gait analysis. PhD thesis]. Jö nkö ping, School of Health Sciences, Jönköping University.

Page, A., Moreno, R., Candelas, P., and Belmar, F., 2008. The accuracy of webcams in 2D motion analysis: sources of error and their control. *European Journal of Physics*, 29 (4), 857–870.

Paton, C.D., 2009. Effects of shoe cleat position on physiology and performance of competitive cyclists. *International Journal of Sports Physiology and Performance*, 4 (4), 517–523.

Priego Quesada, J., Quesada, J.I.P., Jacques, T.C., Bini, R.R., and Carpes, F.P., 2016. Importance of static adjustment of knee angle to determine saddle height in cycling. *Journal of Science and Cycling*, 5 (1), 26–31.

Priego Quesada, J.I., Pérez-Soriano, P., Lucas-Cuevas, A.G., Palmer, R.S., and Anda, R.M.C.O. de, 2017. Effect of bike-fit in the perception of comfort, fatigue and pain. *Journal of Sports Sciences*, 35 (14), 1459–1465.

Salai, M., Brosh, T., Blankstein, A., Oran, A., and Chechik, A., 1999. Effect of changing the saddle angle on the incidence of low back pain in recreational bicyclists. *British Journal of Sports Medicine*, 33 (6), 398–400.

Sanderson, D.J. and Black, A., 2003. The effect of prolonged cycling on pedal forces. *Journal of Sports Sciences*, 21 (3), 191–199.

Steurer, J., 2011. The Delphi method: an efficient procedure to generate knowledge. *Skeletal Radiology*, 40 (8), 959–961.

Too, D., 1994. The effect of trunk angle on power production in cycling. *Research Quarterly for Exercise and Sport*, 65 (4), 308–315.

Umberger, B.R. and Martin, P.E., 2001. Testing the Planar Assumption during Ergometer Cycling. *Journal of Applied Biomechanics*, 17 (1), 55–62.

Van Sickle, J.R. and Hull, M.L., 2007. Is economy of competitive cyclists affected by the anterior–posterior foot position on the pedal? *Journal of Biomechanics*, 40 (6), 1262–1267.

Verma, R., Hansen, E.A., de Zee, M., and Madeleine, P., 2016. Effect of seat positions on discomfort, muscle activation, pressure distribution and pedal force during cycling. *Journal of Electromyography and Kinesiology*, 27, 78–86.

Wanich, T., Hodgkins, C., Columbier, J.-A., Muraski, E., and Kennedy, J.G., 2007. Cycling Injuries of the Lower Extremity. *Journal of the American Academy of Orthopaedic Surgeons*, 15 (12), 748–756.
